# Open questions: what about the ‘other’ Rho GTPases?

**DOI:** 10.1186/s12915-016-0289-7

**Published:** 2016-08-04

**Authors:** Anne J. Ridley

**Affiliations:** Randall Division of Cell and Molecular Biophysics, King’s College London, Guy’s Campus, London, SE1 1UL UK

## Abstract

Rho GTPases have many and diverse roles in cell physiology, and some family members are very well studied, including RhoA, Rac1 and Cdc42. But many are relatively neglected, and fundamental questions about their mechanisms and functions remain open.

Rho GTPases are household names for anyone who works on eukaryotic cell migration and their functions in cell migration, cell division and cell polarity are described in most textbooks on cell biology. Yet most of what we know about Rho GTPases comes from studying a small subset of the many different family members, namely RhoA, Rac1 and Cdc42. There are over 5000 papers related to each of these, whereas members of the second tier of Rho GTPases (for example, Rac2, RhoB and RhoC) have around 200–700 papers each. Humans have 20 different genes encoding Rho GTPases [1] and comparatively little is known about the functions of several of these, including RhoD, RhoF, RhoH, RhoJ, RhoQ, Rnd1, Rnd2, Rnd3, RhoBTB1 and RhoBTB2. Here are three areas where much remains to be discovered about these Rho GTPases.

## How are they regulated?

**Fig. 1:**
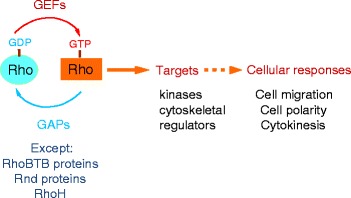
Regulation of Rho GTPases. Most Rho GTPases cycle between an inactive GDP-bound conformation and an active GTP-bound conformation. When bound to GTP, they interact with downstream target proteins to induce cellular responses, for example, cell migration, cell polarity and cytokinesis. They are activated by exchange of GDP for GTP, which is stimulated by guanine nucleotide exchange factors (GEFs). They are inactivated by GTP hydrolysis, which is catalysed by GTPase-accelerating factors (GAPs). Exceptions are the Rho family members RhoBTB proteins (RhoBTB1, RhoBTB2), Rnd proteins (Rnd1, Rnd2, Rnd3) and RhoH, which have amino acid substitutions that prevent GTP hydrolysis and, hence, are constitutively GTP-bound

Classic Rho GTPases switch between an active GTP-bound and inactive GDP-bound form. They are activated by guanine-nucleotide exchange factors (GEFs), which stimulate release of GDP, allowing GTP to bind (Fig. 1), and inactivated by GTPase-accelerating proteins (GAPs), which catalyse GTP hydrolysis, converting them to the GDP-bound form. There are around 80 GEFs and 80 GAPs in humans [2, 3], yet of the ones that have been studied, nearly all have only been tested on RhoA, Rac1 and/or Cdc42. With a few exceptions, we just do not know if they interact with and/or regulate the activity of other family members. Indeed, by studying only RhoA, Rac1 and Cdc42, we are likely to be missing the real functions of many GEFs and GAPs because their targets in cells are among the other Rho GTPases.

Interestingly, four family members—Rnd1, Rnd2, Rnd3 and RhoH—are ‘atypical’, in that they are known to be constitutively GTP-bound and do not hydrolyse GTP: much less is known about how these family members are regulated. RhoU and RhoV have high intrinsic GDP/GTP exchange rates, so are unlikely to need GEFs for activation but could still be turned off by GAPs [1, 4]. For two other members, RhoBTB1 and RhoBTB2, the Rho domains are quite divergent in sequence from other family members and they are unlikely to be regulated by GEFs or GAPs [5].

Information on other mechanisms to modulate Rho GTPases is missing for most of the family. For example, RhoA and Rac1 proteins are targeted for degradation following ubiquitination, but few other members of the family have been tested for ubiquitination [4]. In addition, RhoA and Rac1 have been highly studied for effects of phosphorylation on their activity, but apart from the regulation of Rnd3 by phosphorylation, little is known about how phosphorylation affects other Rho GTPases or even if they are phosphorylated [4].

## How do they signal in cells?

When bound to GTP, Rho GTPases interact with their downstream targets to induce cellular responses (Fig. 1). As described above, post-translational modifications can modulate their activity. Many downstream targets for RhoA, Rac1 and Cdc42 have been identified by a combination of biochemical, molecular and bioinformatics approaches, and structural studies have clearly elucidated the mechanisms of interaction for some [6]. The interactions of other Rho GTPases with known RhoA, Rac1 or Cdc42 targets have been tested individually, but in most cases no concerted approach has been taken to identify their targets in the way that targets have been identified for RhoA, Rac1 and Cdc42. The exception is the Rnd proteins, for which several new targets have been identified by yeast two-hybrid screening, although so far we know relatively little about the functional consequences of these interactions [7]. RhoBTB proteins have been shown to interact via their BTB domains with Cullin-3, a scaffold for ubiquitin ligase complexes, but again the functional role of this interaction is not clear [5]. A challenge for the future will be to identify the protein interactome for each Rho GTPase and to determine how their interactions change dynamically during cellular responses and vary between cell types and with external conditions.

## What are their cellular and organismal functions?

While the effects of RhoA, Rac1 and Cdc42 on cytoskeletal dynamics, cell migration, intracellular membrane transport and cell polarity are well established [8], there are other Rho GTPases for which we have relatively little information about their physiological roles [1]. Some have been more comprehensively studied in specific cell types. For example, Rnd3 has been shown to reduce actomyosin contractility via its interaction with a GAP for RhoA, p190RhoGAP, and, hence, to contribute to migration of mouse neurons in vivo [9]. RhoH is known to contribute to T-cell receptor signalling and, hence, affects T-cell development and function in mice [10]. Studies on the roles of other Rho family members in model organisms will provide new insight into their functions.
